# Burden of psychological symptoms and disorders among individuals with hepatitis B: a systematic review, meta-analysis and meta-regression

**DOI:** 10.3389/fpsyt.2025.1546545

**Published:** 2025-03-24

**Authors:** Chen Ee Low, Genevieve Ge, Trevor James Jun-Ming Yeong, Sounak Rana, Sean Loke, Wei Chieh Kow, Ainsley Ryan Yan Bin Lee, Cyrus Su Hui Ho

**Affiliations:** ^1^ Yong Loo Lin School of Medicine, National University of Singapore, Singapore, Singapore; ^2^ Division of Hepatobiliary and Pancreatic Surgery, Department of Surgery, National University Hospital, Singapore, Singapore; ^3^ Department of Psychological Medicine, Yong Loo Lin School of Medicine, National University of Singapore, Singapore, Singapore; ^4^ Department of Psychological Medicine, National University Hospital, Singapore, Singapore

**Keywords:** hepatitis B, viral infection, mental health, depression, anxiety

## Abstract

**Introduction:**

Hepatitis B is a highly contagious viral infection that has long been a significant global health concern. Given its adverse effects on the course of the disease, evaluating psychiatric outcomes is important. Despite indications of an increased risk of psychological outcomes among those with hepatitis B, the extent of this association remains unclear.

**Methods:**

This PRISMA-adherent systematic review (PROSPERO: CRD42024564246) searched PubMed, Embase, Cochrane, and PsycINFO for all studies evaluating the prevalence and risk of anxiety and depressive symptoms in individuals with hepatitis B. Random effects meta-analyses and meta-regression were used for primary analysis.

**Results:**

A total of 31 studies were included. We identified a high prevalence of depressive symptoms (Proportion=19%, 95% CI: 11-31) and anxiety (Proportion=30%, 95% CI: 18-45) among individuals with hepatitis B. There was also a significantly increased risk of depressive symptoms (RR=1.45, 95% CI: 1.00-2.09, P=0.049) and anxiety (RR=1.40, 95% CI: 1.11-1.78) in individuals with hepatitis B compared to controls. Subgroup analyses indicated that older age and chronic hepatitis B infection were associated with a higher prevalence of anxiety and depressive symptoms. The systematic review found that being single, unemployed, having a lower income, a lower education level, high comorbidities, and a family history of mental illness were significant risk factors for poorer psychological outcomes.

**Conclusion:**

Our study highlights an increased vulnerability to anxiety and depressive symptoms among individuals with hepatitis B. We emphasize the urgent need for early detection and additional support for this at-risk group.

**Systematic review registration:**

https://www.crd.york.ac.uk/prospero/, identifier CRD42024564246.

## Introduction

Hepatitis B, a highly contagious viral infection primarily affecting the liver, has long been a significant global health concern, infecting approximately 400 million people worldwide and resulting in one million deaths annually due to liver disease (1). This persistent viral infection can range from an inactive carrier state to progressive chronic hepatitis B, which may lead to severe liver complications such as cirrhosis and hepatocellular carcinoma, affecting 15-40% of individuals infected with hepatitis B (2).

Beyond these well-documented physical health impacts, there is a growing body of research on the negative psychological outcomes faced by individuals with hepatitis B as a chronic illness. A well-established bidirectional relationship exists between chronic physical illnesses and mental health disorders (3–9). Those with chronic infections such as hepatitis B encounter numerous challenges in managing their illness, ranging from the emotional burden of coping with a long-term condition to the side effects of antiviral therapies and the social stigma associated with their situation (10).

Such factors can significantly contribute to the development of mental health issues and adversely impact the treatment and prognosis of chronic disease. Elevated psychological symptoms, such as depression and anxiety, have been associated with reduced quality of life, poorer medication compliance and increased mortality (11).

In the specific context of hepatitis B, several studies have associated the presence of negative psychological outcomes with higher prevalence rates of psychological disorders among individuals with hepatitis B compared to healthy controls (12). A significant proportion (about 50%) of individuals with hepatitis B and hepatitis C also develop psychiatric illnesses (13). Among these individuals, particularly those with hepatitis B, the global Health-Related Quality of Life (HRQoL) is significantly worse than in the general population, and it appears to decrease as the liver disease advances (14). Notably, in individuals with hepatitis B, depressive symptoms have been associated with a higher risk of liver-related death (15).

Evaluating the psychological outcomes of hepatitis B is therefore important, given their negative impact on the course of the disease. Despite indications of an increased risk of psychological issues among those with hepatitis B, the extent of this association remains unclear, and reported prevalence rates of anxiety and depressive symptoms in individuals with hepatitis B differ across studies (16). Furthermore, only a limited number of studies have examined the prevalence of these symptoms in individuals with hepatitis B infection.

In light of these observations, a comprehensive synthesis of the existing literature is necessary to enhance our understanding of the relationship between hepatitis B and psychological outcomes. Therefore, this study aims to evaluate whether infection heightens the risk of developing psychological issues such as anxiety and depressive symptoms. The objective is to identify potential causes and modifiers of this association and provide a more precise estimate of the mental health burden linked to hepatitis B by integrating data from various research studies. By consolidating available evidence, this study seeks to inform clinical practice and direct future research to improve the comprehensive care of individuals living with hepatitis B.

## Methodology

This systematic review is reported based on the Preferred Reporting Items for Systematic Reviews and Meta-Analyses (PRISMA) guidelines. The protocol was registered on (PROSPERO: CRD42024564246).

### Search strategy

A literature search was performed in PubMed, Embase, Cochrane and PsycINFO. Our search strategy combined terms for ‘hepatitis b’, ‘anxiety’ and ‘depression’. The database-controlled vocabulary was utilized to search for subject headings. Synonyms with appropriate truncations were used to search for title and abstract keywords. The search strategy was translated between the different databases. Examples of the search strategies for PubMed and EMBASE are found in **Supplementary Table S1**. We conducted a hand-searching process and performed both forward and backward searching to ensure inclusion of all relevant articles.

### Inclusion and exclusion criteria

**Figure 1:**
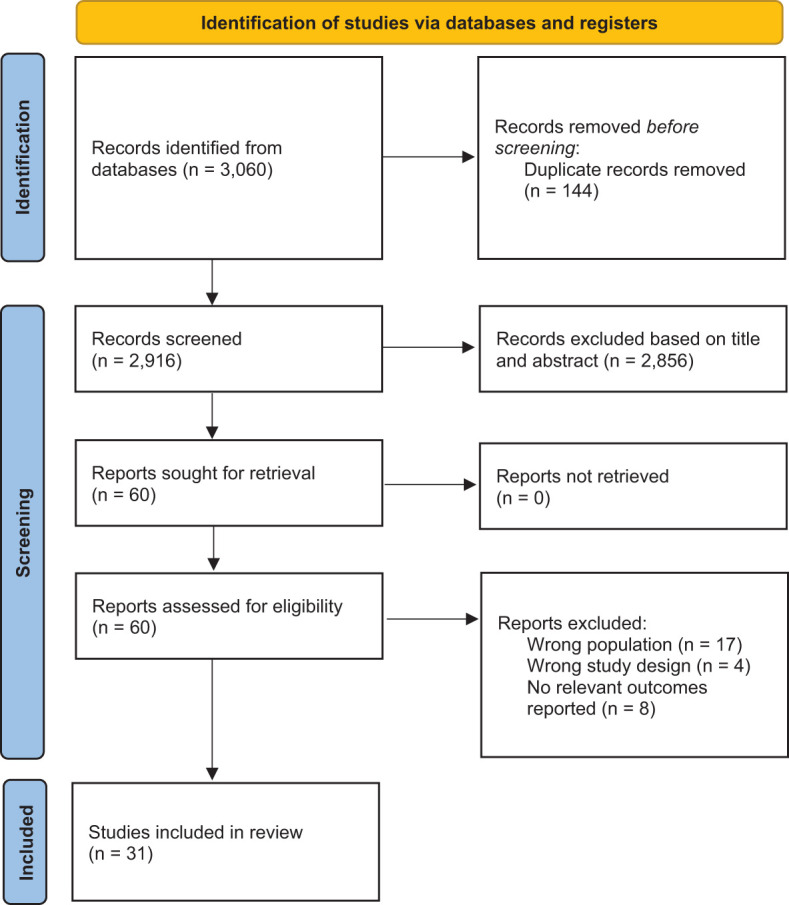
PRISMA Flowchart.

Two reviewers independently screened the titles and abstracts of the studies for eligibility, while a third reviewer addressed any discrepancies. All peer-reviewed studies published in English from 2000 to 2nd November 2024 that assessed the prevalence and risk of psychological outcomes in individuals with hepatitis B were included. The initial extraction date was 1^st^ September 2024. To mitigate the small study effect, only studies with more than 50 individuals with hepatitis B were considered. Non-empirical studies, grey literature, and abstracts were excluded. The selection process is illustrated in **Figure 1**.

### Data extraction and analysis

Two reviewers independently performed the extraction. Subject matter information included demographics, the instruments and scales used to assess psychological outcomes, treatment modalities, chronicity of disease, comorbidities, characteristics of the control group, and the study’s main findings. The number of events and participants at risk regarding the specific outcomes were extracted. We pooled the prevalence for single-arm studies and the relative risk-ratio (RR) for double-arm studies. The reported psychological outcomes must be assessed using a validated instrument for patient-reported outcome measures or a clinical diagnosis made by a registered practitioner.

All analyses were conducted using R (version 4.1.0) along with the *meta* and *metafor* packages. A two-sided P value of <0.05 was deemed statistically significant. Metaprop was employed to meta-analyze the prevalence under a generalized linear mixed model (GLMM). For dichotomous outcomes, meta-analyses were carried out to estimate the relative risk of the psychological outcome (measured using RR compared to controls). We performed sensitivity analyses by identifying and excluding potential outliers, employing random-effects models, and conducting leave-one-out analysis. Subgroup analyses and meta-regression were performed to ascertain whether key hierarchical and categorical variables influenced the results.

I2 and τ2 statistics were used to represent between-study heterogeneity. An I2 of less than 30% indicated low heterogeneity, 30% to 60% suggested moderate heterogeneity and more than 60% demonstrated substantial heterogeneity (17). Egger’s test was used to assess quantitative publication bias (18). Visual inspection of funnel plot asymmetry was conducted to evaluate qualitative publication bias. If publication bias was suspected, a sensitivity analysis was conducted using the trim-and-fill method (R0 estimator and random effects models) to re-estimate the effect size after imputing for potential studies (19). We assumed a normal distribution of effect sizes around the center of the funnel plot if publication bias was absent (18).

### Risk of bias

Two independent reviewers assessed the included studies’ risk of bias using the Joanna Briggs Institute (JBI) Critical Appraisal tool (20). A third reviewer resolved any discrepancies.

## Results

31 studies (21–49) were eventually included from 3,060 records (**Figure 1**). 14 were from Asia (15, 21, 25, 27–31, 34, 42, 45, 46, 49, 50), 10 were from the Middle East (23, 24, 30, 36, 37, 39–41, 48, 51), four were from Europe (22, 33, 43, 47), and three were from North America (35, 38, 44). 53,964 individuals with hepatitis B were included. The mean age of the individuals ranged from 31.6 to 60 years. Most of the studies investigated chronic individuals with hepatitis B, except for two (23, 29). 16 studies (21–26, 29, 31, 32, 34, 37, 40, 42, 43, 47) evaluated both anxiety and depression, 12 studies (15, 27, 28, 30, 33, 35, 38, 39, 41, 44, 45, 50) on depression and three studies (36, 46, 48) on anxiety only. The outcomes from 21 studies (23, 26, 30–32, 34, 36–49) were patient-reported outcome measures, and 10 studies (21, 22, 24, 25, 27–29, 33, 35, 50) with outcomes from a formal clinical diagnosis of depression. Eight studies (22, 24–26, 28–30, 32, 37, 40, 43, 46) were controlled studies. Six studies used healthy individuals from the general population as the comparator group (22, 24, 26, 29, 32, 43). One study used HBsAg-positive inactive carriers as the control group (40). One study used healthy pregnant women as the control group (46). The overall characteristics of the studies can be found in **Table 1**.

**Table 1:** Main characteristics of the included studies.

Author	Publication year	Region of study	Gender (Male) %	Number of individuals with hepatitis B	Number of events*	Age at study (Mean ± SD)	No. of years after hepatitis B diagnosis (Mean ± SD)	Chronicity of Hepatitis B>	Outcomes Investigated	Method of diagnosis (PROM/Clinical Diagnosis)	Instrument, scales, and diagnostic criteria for assessing psychological outcome	Characteristic of controls
Chan (21)	2012	Asia	68	149	48	47.2 (11.9)	>5	Chronic	Anxiety, depressive symptoms	Clinical	Chinese Bilingual Structured Clinical Interview for the Diagnostic and Statistical Manual of Mental Disorders-IV	NA
Cortesi (22)	2020	Europe	53	284	100	60 (12.9)	NR	Chronic	Anxiety, depressive symptoms	Clinical	EQ-5D	Matched
Daryani (23)	2008	Middle East	64	100	35	31.6 (11.6)	<6 months	Acute	Anxiety, depressive symptoms	PROM	CHQ-28	NA
Karlidağ (24)	2019	Middle East	68	197	66	39.01 (11.38)	10.59 (6.1)	Chronic	Anxiety, depressive symptoms	Clinical	HAM-A, HAM-D	Matched
Gupta (20)	2020	Asia	85	150	32	39 (11.9)	72.8 (50.1) months	Chronic	Anxiety, depressive symptoms	Clinical	MINI-PLUS	Inactive carriers
Huang (50)	2019	Asia	67	209	61	44.4 (14.7)	NR	Chronic	Depressive symptoms	Clinical	HAM-D	NA
Kiratli (26)	2023	Middle East	57	118	74	40.76 (16.21)	NR	Chronic	Anxiety, depressive symptoms	PROM	HADS	Healthy
M. Liu (27)	2017	Asia	72	634	41	48 (13.8)	14 (11.4)	Chronic	Depressive symptoms	Clinical	MINI Version 5.0, MADRS	NA
Y. Liu (28)	2017	Asia	52	15558	101	49.57 (10.07)	NR	NR	Depressive symptoms	Clinical	CIDI-SF	Healthy
Chong (29)	2018	Asia	57	34459	3085	43.4 (14.2)	NR	Acute	Anxiety, depressive symptoms	Clinical	NR	Matched
Marcellin (30)	2008	Asia	81	448	13	32.5 (NR)	NR	Chronic	Depressive symptoms	PROM	Frequency of clinically adverse events	Hepatitis C
Ngo (31)	2019	Asia	71	432	115	NR	NR	Chronic	Anxiety, depressive symptoms	PROM	EQ-5D	NA
Saffari (32)	2017	Middle East	77	418	170	44.12 (11.48)	NR	Chronic	Anxiety, depressive symptoms	PROM	EQ-5D	Healthy
Shaheen (33)	2023	Europe	56	1401	126	41.08 (12.64)	NR	Chronic	Depressive symptoms	Clinical	NA	NA
Vu (34)	2019	Asia	55	298	131	49.2 (16.0)	NR	Chronic	Anxiety, depressive symptoms	PROM	PHQ-9	NA
Weinstein (35)	2011	North America	62	190	7	43.6 (13.2)	NR	Chronic	Depressive symptoms	Clinical	NA	NA
Yilmaz (37)	2016	Middle East	46	77	45	36 (9.02)	NR	Chronic	Anxiety, depressive symptoms	PROM	HADS	Healthy
Yılmaz (36)	2022	Middle East	56	505	275	41.2 (17.6)	7.5 (4.6)	Chronic	Anxiety	PROM	NR	NA
Zhu (38)	2022	North America	57	313	129	53.68 (13.42)	21.72 (11.78)	Chronic	Depressive symptoms	PROM	PHQ-9	NA
Ahmed (39)	2017	Middle East	54	67	37	40.58 (9.12)	NR	Chronic	Depressive symptoms	PROM	HADS	NA
Demir (40)	2013	Middle East	60	195	NA	NR	NR	Chronic	Anxiety, depressive symptoms	PROM	HAM-A, HAM-D	Inactive carriers
Alian (41)	2013	Middle East	78	154	150	38.27 (9.52)	12.37 (9.18)	Chronic	Depressive symptoms	PROM	BDI	NA
Chang (42)	2022	Asia	54	215	179	51.9 (11.9)	NR	Chronic	Anxiety, depressive symptoms	PROM	HADS	NA
Cho (15)	2020	Asia	53	10834	42508	39.6 (9.70)	NR	Chronic	Depressive symptoms	PROM	CES-D	NA
Fotos (43)	2018	Europe	55	59	NA	44.85 (14.4)	12.37 (9.18)	Chronic	Anxiety, depressive symptoms	PROM	STAI, BDI	Healthy
Gale (44)	2018	North America	49	354	878	30.12 (7.99)	NR	Chronic	Depressive symptoms	PROM	PHQ-9, CIDI	NA
Huang (45)	2013	Asia	85	73	1	41 (9)	NR	Chronic	Depressive symptoms	PROM	HAM-D	NA
Zhou (46)	2015	Asia	0	160	44	28.13 (3.4)	NR	Chronic	Anxiety	PROM	STAI, PSRS	Healthy
Simonetti (47)	2018	Europe	67	102	72	52 (16)	19 (11)	Chronic	Anxiety, depressive symptoms	PROM	IBQ	NA
Kong	2020	Asia	65	188	134	35.82 (10)	NR	Chronic	Anxiety, depressive symptoms	PROM	DASS21	NA
Keskin (48)	2013	Middle East	48	96	NA	47.53 (13.79)	NR	Chronic	Anxiety	PROM	BAS	NA

NR, Not reported; NA, Not Applicable; SD, Standard Deviation; PROM, Patient Reported Outcome Measures; MDD, Major Depressive Disorder; EQ-5D, EuroQol-5 Dimension; CHQ-28, Child Health Questionnaire parent report short form; HAM-A, Hamilton Anxiety Rating Scale; HAM-D, Hamilton Depression Rating Scale; MINI-PLUS, Mini-International Neuropsychiatric Interview-PLUS; HADS, Hospital Anxiety and Depression Scale; MADRS, Montgomery–Åsberg Depression Rating Scale; CIDI-SF, Composite International Diagnostic Interview Short-Form; PHQ-9, Patient Health Questionnaire-9; BDI, Beck Depression Inventory; CES-D, Center for Epidemiological Studies-Depression; STAI, State-Trait Anxiety Inventory; CIDI, Composite International Diagnostic Interview; PSRS, Perceived Stress Reactivity Scale; IBQ, Illness Behavior Questionnaire; DASS21, Depression Anxiety and Stress Scale 21; BAS, Beck Anxiety Scale.

*Score was reported instead of events.

### Depressive Symptoms

**Figure 2:**
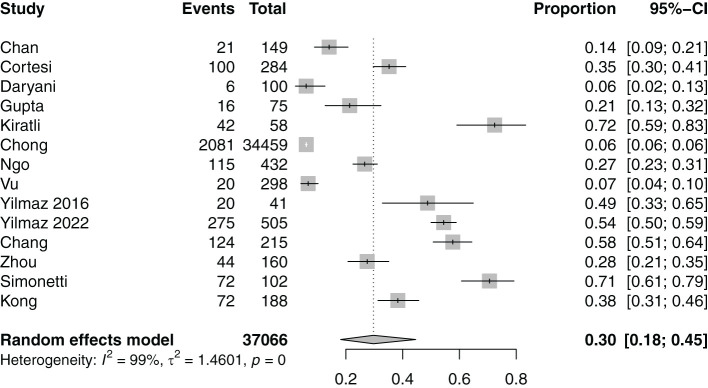
Prevalence of individuals with hepatitis B with depressive symptoms.

23 studies (15, 21–23, 25–31, 34, 35, 37–39, 41, 42, 44, 45, 47, 49, 50) evaluated the prevalence of depressive symptoms among individuals with hepatitis B. Meta-analysis of the 23 studies (15, 21–23, 25–31, 34, 35, 37–39, 41, 42, 44, 45, 47, 49, 50) (**Figure 2**) revealed that depressive symptoms were seen in 19% of individuals with hepatitis B (95%CI: 11-31).

Subgroup analyses of the prevalence of depressive symptoms among other categorical variables are found in **Supplementary Table S2**. Subgroup analyses showed that the prevalence of depressive symptoms was significantly higher in individuals with chronic hepatitis B (15, 21, 22, 25–28, 30, 31, 34, 35, 37–39, 41, 42, 44, 45, 47, 49, 50) (24%, 95%CI: 15-35) as compared to acute hepatitis B (23, 29) (10%, 95%CI: 2-38). The prevalence of depressive symptoms was significantly lower in the clinically assessed group (15, 21, 22, 25, 27–29, 35, 50) (8%, 95% CI: 3-19%) compared to the patient-reported group (23, 26, 30, 31, 34, 37–39, 41, 42, 44, 45, 47, 49) (28%, 95% CI: 17-44%). Studies of populations from the Middle East (23, 30, 37, 39, 41) had the highest prevalence of depressive symptoms (55%, 95%CI: 27-80) and studies from Asia (15, 21, 25, 27–31, 34, 42, 45, 49, 50) had the lowest prevalence of depressive symptoms (11%, 95%CI: 6-19). Subgroup analyses based on the instrument used to assess depressive symptoms were significant. Overall, gender, age at data collection and duration (years) since diagnosis did not significantly increase the prevalence of depressive symptoms. Meta-regression suggested that older age at data collection was significantly associated with the prevalence of depressive symptoms in individuals with hepatitis B (**Supplementary Table S4**).

### Anxiety

**Figure 3:**
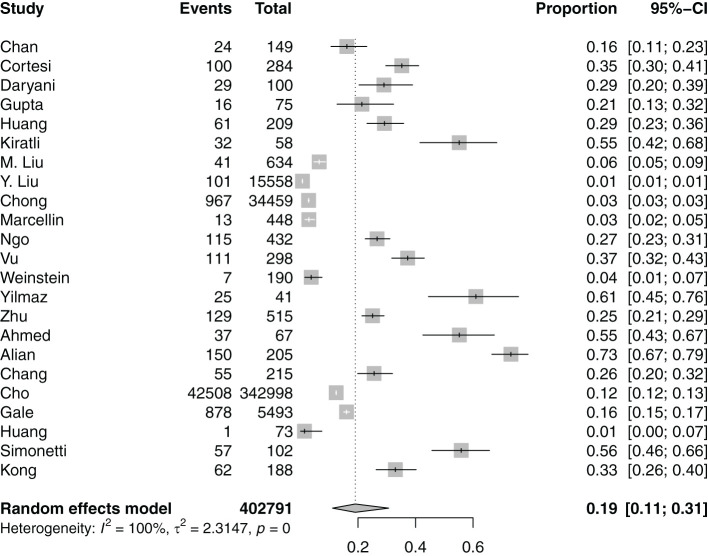
Prevalence of anxiety in individuals with hepatitis B.

14 studies (21–23, 25, 26, 29, 31, 34, 36, 37, 42, 46, 47, 49) evaluated the prevalence of anxiety among individuals with hepatitis B. Meta-analysis of the 14 studies (21–23, 25, 26, 29, 31, 34, 36, 37, 42, 46, 47, 49) (**Figure 3**) revealed that anxiety was observed in 30% of individuals with hepatitis B (95%CI: 18-45).

Subgroup analyses of the prevalence of anxiety among other categorical variables are found in **Supplementary Table S3**. Subgroup analyses showed that anxiety prevalence was significantly higher in individuals with chronic hepatitis B (21, 22, 25, 26, 31, 34, 36, 37, 42, 46, 47, 49) (37%, 95%CI: 25-50) as compared to acute hepatitis B (23, 29) (6%, 95%CI: 2-19). Overall, region, gender, scales used to measure anxiety, outcome measure (clinical diagnosis or patient-reported outcome measure), age at data collection and duration from diagnosis did not significantly increase the prevalence of anxiety. Meta-regression suggested that older age at data collection and duration (years) since diagnosis was significantly associated with anxiety prevalence in individuals with hepatitis B (**Supplementary Table S4**).

**Figure 4:**
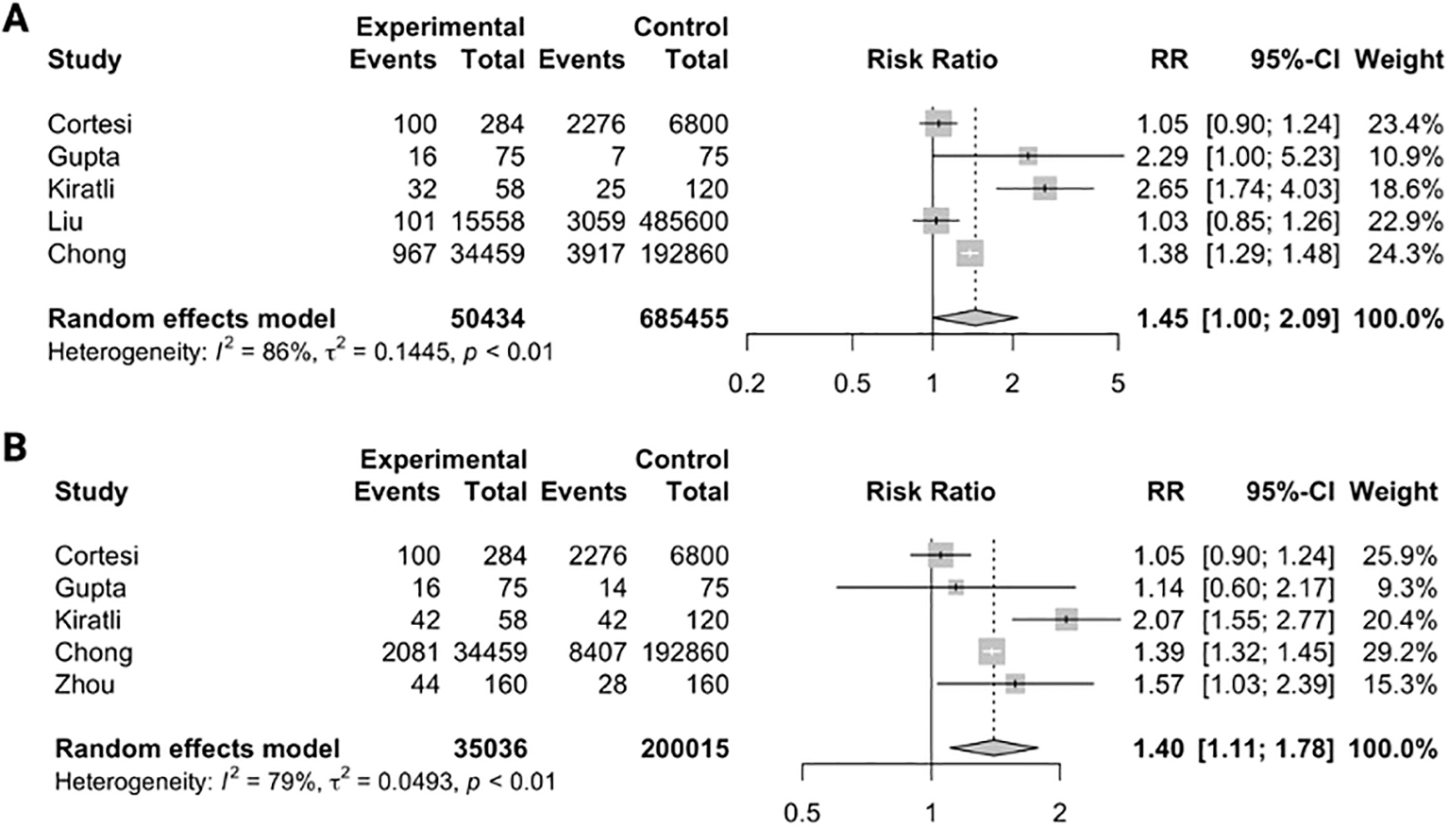
Relative risk ratios of having depressive symptoms **(A)** and anxiety **(B)** in individuals with hepatitis B when compared to comparator.

Five studies (22, 25, 26, 28, 29) were analyzed to investigate the relative risk of depressive symptoms in individuals with hepatitis B. Meta-analysis of the five studies (22, 25, 26, 28, 29) (**Figure 4A**) reported a statistically significant increase in the risk of depressive symptoms in individuals with hepatitis B compared to the comparator arm (RR=1.45, 95%CI: 1.00-2.09, P=0.049). Five (22, 25, 26, 29, 46) studies were analyzed to evaluate the relative risk of anxiety in individuals with hepatitis B. Meta-analysis of the five studies (22, 25, 26, 29, 46) (**Figure 4B**) indicated a statistically significant increased risk of anxiety in individuals with hepatitis B compared to the comparator arm (RR=1.40, 95% CI: 1.11-1.78).

### Risk-of-bias and publication bias

The quality of the methodology of the 31 studies (21–49) was assessed using the JBI checklist and is presented in **Supplementary Table S12**. Some risk of bias was identified. Sensitivity analyses were conducted employing funnel plots, trim-and-fill, and Egger’s test, which indicated some publication bias (**Supplementary Figures S1-S10**). However, the leave-one-out analyses did not reveal any individual studies that would influence the overall results.

### Systematic review

#### Education level

Eight studies (15, 23, 24, 28, 34, 38, 48, 49) reported the confounding effect of education on the psychological outcomes of individuals with hepatitis B (**Supplementary Table S5**). Five (15, 23, 24, 34, 49) out of eight (15, 23, 24, 28, 34, 38, 48, 49) studies reported a significant association between lower education levels and higher levels of depression and anxiety. Three (28, 38, 48) studies found no significant association between education levels and psychological outcomes.

#### Comorbidities

Eight (15, 31, 33, 34, 40, 42–44) studies reported the confounding effect of comorbidities on the psychological outcomes of individuals with hepatitis B (**Supplementary Table S6**). All eight studies (15, 31, 33, 34, 40, 42–44) reported a significant association between having a higher number of comorbidities and a higher likelihood of having depression and anxiety. Interestingly, individuals with alcoholic liver disease (42), liver transplant (31), and herpes simplex virus-2 (44) had worse psychological outcomes.

#### Marital status

Eight (23, 24, 31, 34, 36, 38, 48, 49) studies reported the confounding effect of marital status on the psychiatric outcomes of individuals with hepatitis B (**Supplementary Table S7**). Seven (23, 31, 34, 36, 38, 48, 49) (23, 24, 31, 34, 36, 38, 48, 49) studies showed a significant association between marital status and psychological outcomes. Five (23, 31, 36, 38, 49) out of the seven (23, 31, 34, 36, 38, 48, 49) studies revealed that being single increased the risk of poorer psychological outcomes. Conversely, Vu (34) and Keskin et al (48). reported a significant association between having a spouse, partner or being married and a higher likelihood of worse psychological outcomes.

#### Treatment

Six (29, 40–42, 45, 49) studies reported the confounding effect of treatment factors on the psychological outcomes of individuals with hepatitis B (**Supplementary Table S8**). Kong (49) and Huang et al (45). reported a significant association between a longer duration of treatment and an increased risk of psychological outcomes. Alian et al (41). saw a significantly higher prevalence of depression in participants who received interferon treatment and those who did not. Chang (42) and Demir et al (40). did not observe any significant association between interferon therapy treatment and the prevalence of psychological outcomes. Chong et al (29). found no significant association between antiviral therapy and an increased risk of bipolar disorder.

#### Income

Five (21, 23, 28, 34, 38) studies reported the confounding effect of income on the psychiatric outcomes of individuals with hepatitis B (**Supplementary Table S9**). Four (21, 23, 28, 34) out of five (21, 23, 28, 34, 38) studies reported that Hepatitis B carriers with a lower family income were significantly more likely to have poorer psychological outcomes. Zhu et al (38). did not find any significant relationship between income and depression severity in both treatment and non-treatment groups.

#### Employment status

Three (34, 38, 43) studies reported the confounding effect of employment status on the psychological outcomes of individuals with hepatitis B (**Supplementary Table S10**). Two (38, 43) out of three (34, 38, 43) studies reported a significant association between unemployment and a higher prevalence of worse psychological outcomes. Interestingly, Vu et al (34). found a significant association between freelancers and a lower likelihood of depression.

#### Family history

Three (21, 24, 40) studies reported the confounding effect of family history on psychological outcomes of individuals with hepatitis B (**Supplementary Table S11**). Two (21, 24) out of three (21, 24, 40) studies revealed a significant association between a positive family history of mental illness and worse psychological outcomes. In contrast, Demir et al (40). found no significant association between a family history of Hepatitis B infection or cirrhosis and psychological outcomes.

## Discussion

To the best of our knowledge, this study is the first systematic review and meta-analysis examining the prevalence and risk of anxiety and depressive symptoms among individuals with hepatitis B. Our findings indicated a high prevalence of anxiety and depressive symptoms in those with hepatitis B compared to individuals without the condition. Subgroup analyses identified risk factors for anxiety and depressive symptoms, including older age and chronic hepatitis B infection. The systematic review highlighted that being single, unemployed, having a lower income, lower educational attainment, high comorbidity, and a family history of mental illness were significant risk factors associated with poorer psychological outcomes.

We further compared the prevalence and risk of anxiety and depressive symptoms in individuals with hepatitis B against those with other diseases (**Supplementary Table S15**). Our review found that the prevalence of anxiety and depressive symptoms in individuals with hepatitis B was 30% and 19% respectively, a notably sharp increase compared to the general population, which stands at 4% and 5% respectively (52, 53). Subsequent meta-analysis also revealed a 1.45-fold increase in the risk of depression and a 1.4-fold increase in the risk of anxiety in individuals with hepatitis B compared to the comparator group. The apparent discrepancies in these risk figures highlight the significant psychological burden that may be associated with chronic illness. It is imperative that the healthcare system directs attention not only to treating physical ailments but also to the often-overlooked mental repercussions of hepatitis B.

Furthermore, the prevalence of anxiety among individuals with hepatitis B is higher than that of patients with type 2 diabetes, which is approximately 21.8 (54). The increased prevalence of adverse mental health outcomes in those with hepatitis B may be attributed to the infectious nature and social stigma associated with the condition; whereas diabetes is a non-transmissible disease, hepatitis B can be transmitted through means such as blood, sharing needles, and sexual fluid (55). The concerns that individuals may harbor about the infectiousness of their diagnosis can lead to heightened stress and consequently result in a higher prevalence of psychiatric disorders. Public misconceptions about hepatitis B and its mode of transmission may further amplify the social stigma surrounding these individuals. In a study conducted in Singapore on the general public, over 50% of respondents surveyed believed that hepatitis B could be transmitted by sharing food (56). These misconceptions may result in differential treatment or even avoidance of these individuals, thereby fostering feelings of exclusion or social isolation, which are closely linked to the development of depression (57). Additionally, the prevalence of depression in individuals with hepatitis B is comparable to the prevalence of anxiety among those with asthma, which ranges between 11-13 (58). This similarity further underscores the debilitating impact that chronic disease can have on an individual’s mental health. This aligns with existing literature, which has shown that conditions such as cholesterol disease, kidney disease, coronary heart disease, and asthma are all significantly associated with mental health problems experienced by patients (59).

Subgroup analyses have revealed a significantly higher prevalence of depressive symptoms in individuals with chronic hepatitis B (24%) compared to those with acute hepatitis B (10%). We also observed a notably higher prevalence of anxiety in individuals with chronic hepatitis B (37%) in contrast to acute hepatitis B (6%). These findings are supported by current literature, which demonstrates a well-recognized link between chronic illnesses and mental health disorders (60–62). The discrepancy in our findings may be attributed to biological factors, such as neurotransmitter imbalances associated with chronic illness (63–65). While the stress that infections impose on the immune system triggers cytokine release, which is beneficial in the short term for combating infections, prolonged exposure to inflammatory cytokines can lead to the emergence of neuropsychiatric disorders like depression. Chronic hepatitis B induces ongoing inflammation and activates cell-mediated immunity, which has been shown to play a key role in influencing both the risk of developing depression and its progression (66). Conversely, it has also been noted that individuals experiencing depression and fatigue display increased inflammatory immune activation (67). This underscores the bidirectional relationship between chronic hepatitis B and psychiatric disorders, whereby persistent inflammation not only contributes to depressive symptoms but is also affected by the presence of chronic health conditions.

In addition to biological factors, one of the mainstays of chronic hepatitis B treatment is interferon therapy (68). The adverse side effects of interferon therapy, which are well documented in the literature to include depressive symptoms and anxiety, are plausible contributing factors to our findings (69, 70). Pinto et al (71). describe the prevalence of depression during interferon treatment as 30-70% (72). It has been postulated that interferon-alpha activates the enzyme indoleamine 2,3-dioxygenase (IDO), which shifts tryptophan metabolism away from serotonin production towards the kynurenine pathway (73). This shift ultimately reduces serotonin levels in the brain, a depletion that is widely recognized as a driver of depression (73). Lastly, while acute hepatitis B is curable even without treatment, chronic hepatitis B may never be cured (74). Thus, individuals suffering from the latter could experience intensified feelings of helplessness and other negative emotions that are linked to the development of mental health disorders.

Subgroup analyses have identified older age as a risk factor for a higher prevalence of adverse psychological outcomes for both depressive symptoms and anxiety. Several factors contribute to the increased vulnerability of this group to negative mental health outcomes. Firstly, the natural progression of chronic hepatitis B over time, characterized by repeated cycles of injury, healing, and fibrosis, eventually leads to cirrhosis and even liver cancer (75). Increasing age serves as a predictor for these adverse outcomes in HBeAg-positive individuals (76), such as a heightened risk of developing fibrosis and a reduced likelihood of sustained viral suppression (77, 78). The awareness of being at an increased risk for severe health complications due to age can significantly exacerbate anxiety and depression among older individuals. Furthermore, older adults are more likely to experience multimorbidity than their younger counterparts (79). The physical pain and mental fatigue associated with managing multiple chronic illnesses predispose them to a greater risk of developing depression and anxiety (62, 80). Moreover, older adults face an elevated risk of social isolation and loneliness, arising from factors such as the loss of a partner or loved ones, decreased mobility, sensory impairments, and limited familiarity with technology (81). This isolation can negatively impact mental health outcomes, as numerous studies have underscored a significant connection between social isolation and psychiatric disorders like depression and anxiety (66, 82). These findings highlight the detrimental effects of social isolation on mental health, a connection that is particularly pronounced in the elderly population, given their heightened risk of social isolation.

Our systematic review has also identified lower education levels, lower income and unemployment as causative factors in higher levels of depression and anxiety. Our findings are aligned with the current literature, which consistently demonstrates a strong correlation between lower educational attainment and elevated levels of depression and anxiety (83, 84). The relationship between education, income and employment is closely intertwined and studied extensively. Mirowsky et al (85). have shown that higher educational attainment leads to increasingly stable employment, which provides higher incomes. These greater financial resources contribute to one’s higher socioeconomic status and enable wealth accumulation, allowing greater accessibility to quality healthcare and resources for managing mental wellbeing (86). Education also enhances individuals’ knowledge and skills, which can be utilized to develop coping strategies for regulating mental health (87). Furthermore, individuals from low socioeconomic backgrounds face greater exposure to daily stressors, and the cumulative effects of such stressors are pertinent predictors of poorer mental health outcomes in the long run (88).

An increasing number of comorbidities have been identified as risk factors for anxiety and depression. The relationship between comorbidities and poor psychological outcomes is well documented in scientific literature. Lim et al (89). write that those with chronic health conditions face a higher risk of loneliness and its subsequent health impacts. In alignment with this, Ronaldson et al (90). found that individuals with more than one physical health condition tended to have worse mental health than their physically healthier counterparts. The debilitating diagnosis of a chronic illness may threaten an individual’s sense of purpose and meaning in life (91). Individuals with chronic illnesses are also more likely to self-inflict social isolation, as they may perceive themselves as different compared to their healthy counterparts, thus reducing their contact and participation in social activities (51, 92). This is confounded by their disabling symptoms and related discomfort, or they may struggle to engage in social activities due to a lack of energy. Consequently, chronically ill individuals may find themselves lacking emotional support from a healthy social circle, placing them at a heightened risk of developing anxiety and depression.

We have also identified a family history of mental illness as a risk factor for anxiety and depression. This is no surprise, as numerous reputable studies have demonstrated similar findings. Streit et al (93). showed that a family history is linked to increased depression. It was also found that students with a family history of mental illnesses were more likely to report clinically significant depressive symptoms (94). There is growing evidence of the genetic vulnerability involved in developing adverse mental health outcomes. For example, Sullivan et al (95). showed that there is a two-to-three-fold increase in the risk of depression among first-degree relatives of individuals with depression. These conclusions suggest that there is validity in the assertion that a family history of mental illness is a risk factor for anxiety and depression.

Finally, we observed that being married offers a protective advantage against the risk of developing anxiety and depression. The social support provided by marriage exerts far stronger effects than support from friends (96). Thoits et al. (97). suggest that social support is most beneficial when it comes from individuals who share similar characteristics and values and have successfully navigated similar challenges. Hence, the spouse often serves as the pivotal source of support due to the interconnectedness of their relationship with the individual. Furthermore, spousal support is not only unique and sometimes more effective than other forms of support, but in times of crisis, the spouse is often the first person to whom individuals turn for help (98, 99). The presence of such social support fosters a safe environment for individuals to share their feelings and process their emotions, thereby bolstering their mental well-being (100). Social support also positively influences health outcomes by reducing mood-related symptoms and increasing help-seeking behavior and treatment compliance (101). Like many chronic diseases, hepatitis B is associated with a significant impact on mental health (8, 102–104). By identifying and elucidating the psychological burden amongst individuals with hepatitis B, further interventions may be instituted to alleviate this burden (105–107).

### Limitations

Our study should be interpreted with due consideration of certain limitations. Firstly, there was high heterogeneity in the reporting of outcomes that we were unable to account for, such as the variety of instruments used to evaluate depressive symptoms and anxiety among the studies. There were also insufficient studies with a control arm, and among those studies that did include a control arm, data on anxiety and depressive symptoms were available for five studies each. Due to the heterogeneity across different social contexts, we could not perform subgroup meta-analyses for variables such as education level, comorbidities, marital status, treatment factors, income level, employment status and family history. Nonetheless, we were still able to systematically review those factors. Secondly, we did not obtain individual patient data for our meta-analysis as the available data was insufficient to study individual risk factors affecting psychological outcomes. However, we could still analyze the key characteristics of the subgroups we planned for, including age, gender, years since hepatitis diagnosis, chronicity, outcome measure, and region. Thirdly, a large proportion of our studies originate from Asia and the Middle East, with a small proportion from the US and Europe. This may limit the generalizability of these findings to the Caucasian population. However, it is known that the burden of Hepatitis B in Asia far exceeds that in the US, Europe and Oceania (108), which reflects the higher number of studies conducted in Asia. It is well-established that individuals with psychiatric comorbidities are less likely to adhere to treatment regimens for both their chronic illnesses and mental health conditions, leading to adverse health outcomes (109). We hence emphasize the need for early detection and intervention for mental health comorbidities in individuals with hepatitis B.

## Conclusion

Our study highlights an increased susceptibility to anxiety and depressive symptoms in individuals with hepatitis B. Notably, factors such as older age, chronicity, being single, unemployed, having a lower income, a lower education level, high comorbidities, and a family history of mental illness are significant contributors to adverse psychological outcomes. Providing tailored mental health support for this vulnerable group can enhance treatment adherence, mitigate the disease burden, and improve overall well-being. Urgent interventions and further research are necessary to elucidate additional prognostic factors to devise targeted strategies to alleviate the burden of anxiety and depressive symptoms in these individuals. Future research could focus on developing a standardized assessment of mental health in individuals with hepatitis B to identify those at risk of psychological distress. This would facilitate the provision of targeted psychosocial support, potentially improving health and treatment outcomes for these individuals.

## Data Availability

The original contributions presented in the study are included in the article/**Supplementary Material**. Further inquiries can be directed to the corresponding author.
