# Supplementary material for: Burden of psychological symptoms and disorders among individuals with hepatitis B: a systematic review, meta-analysis and meta-regression
Source: Front Psychiatry. 2025 Mar 24;16:1546545. doi: 10.3389/fpsyt.2025.1546545 (PMC11973283; doi:10.3389/fpsyt.2025.1546545)
Supplement: Supplementary file 1 [file SupplementaryFile1.docx]

**Burden of psychological symptoms and disorders among individuals with hepatitis B: A systematic review, meta-analysis and meta-regression**

Supplementary Table 1: Search strategy

Supplementary Table 2: Subgroup meta-analyses of hepatitis B infection and prevalence of depressive symptoms using the random effect model

Supplementary Table 3: Subgroup meta-analyses of hepatitis B infection and prevalence of anxiety using the random effect model

Supplementary Table 4: Mixed effects meta-regression of hepatitis B infection against potential effect moderators (continuous and categorical study-level characteristics) for depressive symptoms and anxiety outcomes

Supplementary Table 5: Evaluation of the mediating or confounding effect of education level on psychological outcomes of Hepatitis B individuals

Supplementary Table 6: Evaluation of the mediating or confounding effect of comorbidities on psychological outcomes of Hepatitis B individuals

Supplementary Table 7: Evaluation of the mediating or confounding effect of marital status on psychological outcomes of Hepatitis B individuals

Supplementary Table 8: Evaluation of the mediating or confounding effect of treatment factors on psychological outcomes of Hepatitis B individuals
Supplementary Table 9: Evaluation of the mediating or confounding effect of income on psychological outcomes of Hepatitis B individuals

Supplementary Table 10: Evaluation of the mediating or confounding effect of employment status on psychological outcomes of Hepatitis B individuals

Supplementary Table 11: Evaluation of the mediating or confounding effect of family history on psychological outcomes of Hepatitis B individuals

Supplementary Table 12: Quality assessment of included cohort studies using the Joanna Brigg’s Institute Critical Appraisal tool

Supplementary Table 13: Comparison of anxiety and depressive symptoms among individuals of different chronic diseases

Supplementary Figure 1: Funnel plot for visual inspection of publication bias in studies assessing depressive symptoms prevalence in individuals with hepatitis B infection

Supplementary Figure 2: Trim-and-fill analysis for publication bias in studies assessing depressive symptoms prevalence in individuals with hepatitis B infection

Supplementary Figure 3: Quantitative assessment publication bias in studies assessing depressive symptoms prevalence in individuals with hepatitis B infection

Supplementary Figure 4: Outlier assessment of studies assessing depressive symptoms prevalence in individuals with hepatitis B infection using the random effects model

Supplementary Figure 5: Leave-one-out analysis of studies assessing depressive symptoms prevalence in individuals with hepatitis B infection using the random effects model

Supplementary Figure 6: Funnel plot for visual inspection of publication bias in studies assessing anxiety prevalence in individuals with hepatitis B infection

Supplementary Figure 7: Trim-and-fill analysis for publication bias in studies assessing anxiety prevalence in individuals with hepatitis B infection

Supplementary Figure 8: Quantitative assessment publication bias in studies assessing anxiety prevalence in individuals with hepatitis B infection

Supplementary Figure 9: Outlier assessment of studies assessing anxiety prevalence in individuals with hepatitis B infection using the random effects model

Supplementary Figure 10: Leave-one-out analysis of studies assessing anxiety prevalence in individuals with hepatitis B infection using the random effects model

Search strategy

**EMBASE**

| #1 | ('hepatitis b'/exp OR 'hbv infection':ti,ab OR 'hepatitis b infection':ti,ab OR 'hepatitis b viral infection':ti,ab OR 'hepatitis b virus infection':ti,ab OR 'infection by hbv':ti,ab OR 'infection by hepatitis b virus':ti,ab OR 'infection caused by hepatitis b virus':ti,ab OR 'type b hepatitis':ti,ab OR 'viral hepatitis b':ti,ab OR 'viral hepatitis type b':ti,ab OR 'virus hepatitis type b':ti,ab OR 'hepatitis b':ti,ab) |
| --- | --- |
| #2 | ('depression' OR 'anxiety' OR 'anxiety disorder' OR 'posttraumatic stress disorder' OR ‘suicide’ OR ‘suicide ideation’ OR ‘schizophrenia’ OR ‘psychosis’)/exp OR ‘depressive’ OR 'depress*' OR 'anxi*' OR 'ptsd' OR 'post-traumatic' OR 'post traumatic' OR 'traumatic stress*' OR 'suicidal behavior' OR ‘suicid*’ OR ‘schizo*’ OR ‘psychotic’ OR ‘psychoses’):ab,ti |

#1 and #2
Limited to Year 2000>

**PubMed**

| #1 | "Hepatitis B"[Mesh] OR "Hepatitis B Virus Infection"[Title/Abstract] OR 'hbv infection'[Title/Abstract] OR 'hepatitis b infection'[Title/Abstract] OR 'viral hepatitis b'[Title/Abstract] |
| --- | --- |
| #2 | (“Depression”[Mesh] OR “Anxiety Disorders”[Mesh] OR "Suicide"[Mesh] OR "Schizophrenia"[Mesh] OR "Stress Disorders, Post-Traumatic"[Mesh] OR "Depressive*"[tiab] OR "Anxi*"[tiab] OR "Suicid*"[tiab] OR "Post-Traumatic*"[tiab] OR "PTSD"[tiab] OR "posttraumatic*"[tiab] OR "Post Traumatic*"[tiab] OR “Disorder*, Schizophrenic”[tiab] OR “Schizo*”[tiab] OR “Psychotic”[tiab] OR “Psychosis”[tiab]) |

#1 and #2
Limited to Year 2000>

Supplementary Table 2: Subgroup meta-analyses of hepatitis B infection and prevalence of depressive symptoms using the random effect model

| **Variable** | **Cohorts** | **Number at risk** | **Proportion** | **95% CI** | **I2** | **Test of interaction (p-value)** |
| --- | --- | --- | --- | --- | --- | --- |
| Overall | 23 | 402791 | 0.19 | 0.11; 0.31 | 100% | NA |
| Gender <50% Male | 3 | 5683 | 0.27 | 0.06; 0.68 | 95.3% | 0.56 |
| Gender >50% Male | 20 | 397108 | 0.18 | 0.10; 0.30 | 99.6% |  |
| Age at data collection > 50 | 17 | 401034 | 0.15 | 0.08; 0.27 | 99.6% | 0.39 |
| Ag at data collection < 50 | 5 | 1325 | 0.33 | 0.12; 0.65 | 90.2% |  |
| Age at data collection = NR | 1 | 432 | 0.27 | 0.02; 0.86 | NR |  |
| Years since Hep B diagnosis <10 | 5 | 596 | 0.37 | 0.14; 0.67 | 96.9% | 0.18 |
| Years since Hep B diagnosis >10 | 4 | 1460 | 0.25 | 0.08; 0.57 | 97.9% |  |
| Years since Hep B diagnosis = NR | 14 | 400735 | 0.13 | 0.07; 0.25 | 99.7% |  |
| Chronicity of Disease = CHB | 20 | 352674 | 0.24 | 0.15; 0.35 | 98.6% | **0.007** |
| Chronicity of Disease = Acute | 2 | 34559 | 0.10 | 0.02; 0.38 | 99.3% |  |
| Chronicity of Disease = NR | 1 | 15558 | 0.007 | 0.006; 0.07 | NR |  |
| Outcome = Clinical | 8 | 51558 | 0.08 | 0.03; 0.19 | 99.4% | **0.0142** |
| Outcome = Patient Reported Outcome | 15 | 351233 | 0.28 | 0.17; 0.44 | 98.8% |  |
| Scale = NR | 3 | 34798 | 0.05 | 0.03; 0.09 | 97.2% | **< 0.0001** |
| Scale = EQ-5D | 2 | 716 | 0.31 | 0.19; 0.45 | 83.3% |  |
| Scale = CHQ28 | 1 | 100 | 0.29 | 0.13; 0.51 | NR |  |
| Scale = Mini 5.0 | 2 | 709 | 0.11 | 0.06; 0.20 | 94.4% |  |
| Scale = HADS | 4 | 381 | 0.47 | 0.35; 0.59 | 91.8% |  |
| Scale = CIDI-SF | 1 | 15558 | 0.007 | 0.003; 0.02 | NR |  |
| Scale = Frequency of events | 1 | 448 | 0.03 | 0.01; 0.07 | NR |  |
| SCALE = PHQ-9 | 3 | 6306 | 0.25 | 0.17; 0.35 | 98.0% |  |
| Scale = BDI | 1 | 205 | 0.73 | 0.52; 0.87 | NR |  |
| Scale = CES-D | 1 | 342998 | 0.12 | 0.06; 0.25 | NR |  |
| Scale = HAM-D | 2 | 282 | 0.13 | 0.06; 0.27 | 91.0% |  |
| Scale = IBQ | 1 | 102 | 0.56 | 0.33; 0.77 | NR |  |
| Scale = DASS21 | 1 | 188 | 0.33 | 0.16; 0.55 | NR |  |
| Region = Asia | 13 | 395736 | 0.11 | 0.06; 0.18 | 99.7% | **0.0005** |
| Region = Europe | 2 | 386 | 0.45 | 0.15; 0.80 | 92.3% |  |
| Region = Middle East | 5 | 471 | 0.55 | 0.31; 0.77 | 92.0% |  |
| Region = North America | 3 | 6198 | 0.12 | 0.04; 0.34 | 95.6% |  |

Abbreviations: CHB = Chronic Hepatitis B; NR = No Record; EQ-5D = EuroQol-5 Dimension; CHQ28=Child Health Questionnaire parent report short form; MINI-PLUS = Mini-International Neuropsychiatric Interview 5.0; HADS = Hospital Anxiety and Depression Scale; CIDI-SF = Composite International Diagnostic Interview Short-Form; PHQ-9 = Patient Health Questionnaire-9; BDI = Beck Depression Inventory; CES-D = Center for Epidemiological Studies-Depression; HAM-D = Hamilton Depression Rating Scale; IBQ = Illness Behavior Questionnaire; DASS21 = Depression Anxiety and Stress Scale 21;

Supplementary Table 3: Subgroup meta-analyses of hepatitis B infection and prevalence of anxiety using the random effect model

| **Variable** | **Cohorts** | **Number at risk** | **Proportion** | **95% CI** | **I2** | **Test of interaction (p-value)** |
| --- | --- | --- | --- | --- | --- | --- |
| Overall | 14 | 37066 | 0.30 | 0.18; 0.45 | 100% | NA |
| Age at data collection > 50 years | 10 | 36033 | 0.24 | 0.14; 0.38 | 99.4% | 0.16 |
| Age at data collection < 50 years | 3 | 601 | 0.55 | 0.26; 0.80 | 95.6% |  |
| Age at data collection = NR | 1 | 432 | 0.27 | 0.04; 0.75 | NR |  |
| Years since Hep B diagnosis <10 | 4 | 829 | 0.20 | 0.08; 0.42 | 97.4% | 0.18 |
| Years since Hep B diagnosis >10 | 1 | 102 | 0.71 | 0.18; 0.47 | NR |  |
| Years since Hep B diagnosis = NR | 9 | 36135 | 0.31 | 0.22; 0.95 | 99.4% |  |
| Chronicity of Disease = CHB | 12 | 2507 | 0.37 | 0.25; 0.50 | 96.4% | **0.003** |
| Chronicity of Disease = Acute | 2 | 34559 | 0.06 | 0.02; 0.19 | 0.0% |  |
| Gender <50% Male | 3 | 350 | 0.27 | 0.09; 0.61 | 90.43% | 0.88 |
| Gender >50% Male | 11 | 36716 | 0.30 | 0.17; 0.47 | 99.6% |  |
| Outcome = Clinical | 4 | 34967 | 0.16 | 0.06; 0.37 | 99.1% | 0.0999 |
| Outcome = Patient Reported Outcome | 10 | 2099 | 0.37 | 0.22; 0.54 | 96.9% |  |
| Scale = NA | 2 | 34608 | 0.06 | 0.06; 0.06 | 93.6% | 0 |
| Scale = EQ-5D | 2 | 716 | 0.30 | 0.27; 0.33 | 83.3% |  |
| Scale = CHQ28 | 1 | 100 | 0.06 | 0.03; 0.13 | NR |  |
| Scale = MINI PLUS | 1 | 75 | 0.21 | 0.14; 0.32 | NR |  |
| Scale = HADS | 4 | 819 | 0.56 | 0.53; 0.60 | 60.8% |  |
| SCALE = PHQ-9 | 1 | 298 | 0.07 | 0.04; 0.10 | NR |  |
| Scale = STAI | 1 | 160 | 0.28 | 0.21; 0.35 | NR |  |
| Scale = IBQ | 1 | 102 | 0.71 | 0.61; 0.79 | NR |  |
| Scale = DASS21 | 1 | 188 | 0.38 | 0.32; 0.45 | NR |  |
| Region = Asia | 8 | 35976 | 0.21 | 0.11; 0.36 | 99.3% | 0.1314 |
| Region = Europe | 2 | 386 | 0.53 | 0.21; 0.83 | 97.2% |  |
| Region = Middle East | 4 | 704 | 0.41 | 0.19; 0.67 | 94.6% |  |

Abbreviations: CHB = Chronic Hepatitis B; NR = No Record; EQ-5D = EuroQol-5 Dimension; CHQ28=Child Health Questionnaire parent report short form; MINI-PLUS = Mini-International Neuropsychiatric Interview 5.0; HADS = Hospital Anxiety and Depression Scale; CIDI-SF = Composite International Diagnostic Interview Short-Form; PHQ-9 = Patient Health Questionnaire-9; BDI = Beck Depression Inventory; CES-D = Center for Epidemiological Studies-Depression; HAM-D = Hamilton Depression Rating Scale; IBQ = Illness Behavior Questionnaire; DASS21 = Depression Anxiety and Stress Scale 21;

Supplementary Table 4: Mixed effects meta-regression of hepatitis B infection against potential effect moderators (continuous and categorical study-level characteristics) for depressive symptoms and anxiety outcomes

|  | **Ratio** | **P** | **95% CI Lower** | **95% CI Upper** | **I^2^ (% residual heterogeneity)** |
| --- | --- | --- | --- | --- | --- |
| **Depression** | | | | | |
| Gender>50% | 0.8833 | 0.2710 | -2.7034 | 0.7590 | 99.7% |
| Age at data collection>50 | 0.3603 | **<0.001** | -2.4049 | -0.9927 | 99.8% |
| Years since Hep B diagnosis>10 | 0.6409 | 0.4081 | -1.7863 | 0.7259 | 99.8% |
| **Anxiety** | | | | | |
| Gender>50% | 0.7114 | 0.1773 | -2.3540 | 0.4345 | 99.0% |
| Age at data collection>50 | 0.3470 | **0.0008** | -1.8460 | -0.4858 | 98.4% |
| Years since Hep B diagnosis>10 | 0.5576 | **0.0119** | -2.4947 | -0.3088 | 98.6% |

Abbreviations: CI, confidence interval

Supplementary Table 5: Evaluation of the mediating or confounding effect of education level on psychological outcomes of Hepatitis B individuals

| **Author** | **Year** | **Country** | **Study population** | **Key findings†** |
| --- | --- | --- | --- | --- |
| Daryani | 2008 | Iran | 100 Hepatitis B carriers, mean age 31.6, SD 11.6 years, were recruited for a cross-sectional study in Iran from 2004-2005. | Hepatitis B carriers with only a secondary or high school education were significantly more likely to have anxiety (p=<0.01), depression (p=<0.01) and abnormal psychiatric health (p=<0.001). |
| Karlidag | 2019 | Turkey | 103 individuals with chronic hepatitis B treated with oral antivirals, mean age 39.0, SD 11.4 years, 94 untreated individuals with chronic hepatitis B, mean age 38.5, SD 12.1 years and 50 healthy individuals, mean age 36.6, SD 8.16, were recruited for a case-control study in Turkey in 2018. | As education level decreases, both anxiety (p <0.001) and depression scores (p=0.002) increase significantly. |
| Liu | 2017 | China | 501,158 participants, of whom, 15558 had CHB were recruited for a cross-sectional study in China from 2004 to 2008. | No association was found between education status and major depression among people with different HBV status. |
| Cho | 2020 | Korea | 342,998 participants, mean age 39.6, SD 9.7, consisting of 10,834 HBsAg positive participants were recruited for a cross-sectional study in Korea. | Participants with a lower education level positively associated with lower mental health outcomes. |
| Kong | 2020 | China | 188 participants, mean age 35.82, SD 10, were recruited for a cross-sectional study in China from March to October 2018. | Lower education levels were found to be a risk factor for depression symptoms (aOR = 0.19, 95% CI: 0.04–0.91) |
| Keskin | 2013 | Turkey | 96 participants, mean age 47.53, SD 13.79, were recruited for a cross-sectional study in Turkey between April and September 2008. | Anxiety and depression levels did not differ by education level (p >0.05). |
| Vu | 2019 | Vietnam | 298 participants, mean age 49.2, SD 16.0 were recruited for a cross-sectional study at the Chronic Hepatitis Clinic in the Viet-Tiep Hospital, Hai Phong, Vietnam. | Participants with an educational level past high school (Coef. = -1.64; 95% CI = -2.90–0.37) were negatively correlated to PHQ-9 score. |
| Zhu | 2022 | USA | 313 Asian American participants, 177 not on antiviral medication, mean age 54, SD 13.42, and 136 on antiviral medication, mean age 53.08, SD 13.05, were recruited for a cross-sectional study in the United States between April 2019 and March 2020. | There was no significant association between education and risk for mild or severe depression, both among those not on medication (OR = 1.06, 95% CI 0.42-2.67) and those on medication (OR = 1.94, 95% CI 0.51-7.35). |

Abbreviations: SD, standard deviation; OR, Odds ratio

†Outcomes of interest include logistic or linear regression analysis for any association between education level and psychological outcomes

Supplementary Table 6: Evaluation of the mediating or confounding effect of comorbidities on psychological outcomes of Hepatitis B individuals

| **Author** | **Year** | **Country** | **Study population** | **Key findings†** |
| --- | --- | --- | --- | --- |
| Ngo | 2019 | Vietnam | Cross-sectional study of 432 individuals in Vietnam at different stages of chronic hepatitis B on their quality of life | Asymptomatic individuals were significantly more likely to have better health-related quality of life (score = 0.57) than post-liver transplant individuals (score = 0.51). |
| Vu | 2019 | Vietnam | 298 participants, mean age 49.2, SD 16.0 were recruited for a cross-sectional study at the Chronic Hepatitis Clinic in the Viet-Tiep Hospital, Hai Phong, Vietnam | Participants having a higher number of comorbidities were associated with a higher likelihood of having depressive symptoms (OR = 1.84; 95% CI = 1.17–2.88). |
| Demir | 2013 | Turkey | 444 participants of which 249 HBsAg-positive inactive carriers (inactive carrier group) and 195 chronic hepatitis B individuals (chronic group) that were undergoing follow-up were recruited for a cross-sectional study in Turkey. | Participants with comorbid illnesses in both the inactive carrier and chronic hepatitis B groups were associated with higher anxiety (Hamilton Anxiety Rating Scale) (P = 0.005 and P = 0.001, respectively) than those participants without comorbid illnesses.  Participants in the inactive carrier group with comorbid illness were associated with higher depression scores (Hamilton Depression Rating Scale) (P = 0.003) than those participants without comorbid illnesses. |
| Chang | 2022 | Taiwan | 2537 participants, mean age 51.9, SD 11.9 were recruited for a cross-sectional study in Taiwan from January 2015 to February 2018. | Participants with alcoholic liver disease were significantly more likely to have a higher risk of anxiety (adjusted odds ratio [OR] = 1.83, P <.001) and depression (adjusted OR= 1.85, P < .001).  Participants with fatty liver disease had a marginal association for greater risks of depression (adjusted OR = 1.26, P = .091). |
| Cho | 2020 | Korea | 342,998 participants, mean age 39.6, SD 9.7, consisting of 10,834 HBsAg positive participants were recruited for a cross-sectional study in Korea. | Participants with obesity, diabetes, hypertension, slightly elevated levels of blood glucose and triglycerides were positively associated with depressive symptoms (p<0.001). |
| Fotos | 2018 | Greece | 111 participants, mean age 44.85, SD 14.4, were recruited for a single center cross-sectional study in Greece from March to September of 2014. | Participants with comorbidities were significantly associated with presence of depression (p<0.05). |
| Gale | 2018 | US | 1772 participants, mean age 30.12, SD 8.00 gathered from 1999 to 2004, and 5493 participants, mean age 34.74, SD 16.3 from 2005 to 2012 were recruited for a cross-sectional study in the United States. | Participants with Herpes Simplex Virus-2 were significantly associated with an increased odds of depression (OR = 2.07, p = .01). |
| Shaheen | 2023 | UK | 1401 Hepatitis B positive participants, 1275 no major depressive disorder, mean age 37.41, 14.23, 126 with major depressive disorder, mean age 41.08, SD 12.64 were recruited for a population-based cohort study in the United Kingdom between 1986 and 2017. | Participants with major depressive disorder were associated with at least 1 comorbidity (30.1% vs. 17.6%, p<0.001). |

Abbreviations: SD, standard deviation; OR, odds ratio; CI, Confidence interval
†Outcomes of interest include logistic or linear regression analysis for any association between comorbidities and psychological outcomes

Supplementary Table 7: Evaluation of the mediating or confounding effect of marital status on psychological outcomes of Hepatitis B individuals

| **Author** | **Year** | **Country** | **Study population** | **Key findings†** |
| --- | --- | --- | --- | --- |
| Daryani | 2008 | Iran | 100 hepatitis B carriers, mean age 31.6, SD 11.6 years, were recruited for a cross-sectional study in Iran from 2004-2005. | Widowed hepatitis B carriers were significantly more likely to have depression (p<0.001). |
| Karlidag | 2019 | Turkey | 103 individuals with chronic hepatitis B treated with oral antivirals, mean age 39.0, SD 11.4 years, 94 untreated individuals with chronic hepatitis B, mean age 38.5, SD 12.1 years and 50 healthy individuals, mean age 36.6, SD 8.16, were recruited for a case-control study in Turkey in 2018. | No significant relation was detected between marital status and score of anxiety and depression (p>0.05). |
| Kong | 2020 | China | 188 participants, mean age 35.82, SD 10, were recruited for a cross-sectional study in China from March to October 2018. | Participants with an unmarried status (aOR = 3.48, 95% CI: 1.25–9.72) were more likely to suffer from stress symptoms. |
| Keskin | 2013 | Turkey | 96 participants, mean age 47.53, SD 13.79, were recruited for a cross-sectional study in Turkey between April and September 2008. | There was a significant difference between depression and marital status (p<0.05). Depression levels were found to be “severe” in married individuals, whereas it was “mild” in single or divorced individuals.  There was no significant difference between anxiety levels and marital status. |
| Ngo | 2019 | Vietnam | Cross-sectional study of 432 individuals in Vietnam at different stages of chronic hepatitis B on their quality of life | The study found that there was significantly lower HRQoL scores in individuals who are single. |
| Zhu | 2022 | USA | 313 Asian American participants, 177 not on antiviral medication, mean age 54, SD 13.42, and 136 on antiviral medication, mean age 53.08, SD 13.05, were recruited for a cross-sectional study in the United States between April 2019 and March 2020. | Among those not on medication, being currently married (OR = 0.30, p < 0.05) was associated with a lower risk for depression. |
| Vu | 2019 | Vietnam | 298 participants, mean age 49.2, SD 16.0 were recruited for a cross-sectional study at the Chronic Hepatitis Clinic in the Viet-Tiep Hospital, Hai Phong, Vietnam. | Individuals who had a spouse or partner (OR = 3.66; 95%CI = 0.95;14.13) were associated with a higher likelihood of having depressive symptoms  Individuals having a spouse/partner (Coef. = 2.35; 95%CI = 0.44–4.26) were positively correlated to PHQ-9 score. |
| Yilmaz | 2022 | Turkey | 505 participants, mean age, 41.2, SD 17.6, were recruited for a cross-sectional study in Turkey between 1 January and 31 December 2015. | Those who had never been married were more inclined to hide their hepatitis positivity (P=0.020) |

Abbreviations: SD, standard deviation; OR, Odds ratio; CI, Confidence interval; ​​HRQoL, Health Related Quality of Life

†Outcomes of interest include logistic or linear regression analysis for any association between marital status and psychological outcomes

Supplementary Table 8: Evaluation of the mediating or confounding effect of treatment factors on psychological outcomes of Hepatitis B individuals

| **Author** | **Year** | **Country** | **Study population** | **Key findings†** |
| --- | --- | --- | --- | --- |
| Demir | 2013 | Turkey | 444 participants of which 249 HBsAg-positive inactive carriers (inactive carrier group) and 195 chronic hepatitis B individuals (chronic group) that were undergoing follow-up were recruited for a cross-sectional study in Turkey. | There was no significant difference in anxiety or depression scores based on interferon use (p>0.05). |
| Kong | 2020 | China | 188 participants, mean age 35.82, SD 10, were recruited for a cross-sectional study in China from March to October 2018. | Participants with a longer duration of treatment were significantly associated with an increased risk of anxiety symptoms (aOR = 2.29, 95% CI: 1.02–5.15). |
| Alian | 2013 | Iran | 205 participants, mean age 38.27, SD 9.52, were recruited for a cross-sectional study in Iran. | The prevalence of depression was significantly higher in participants on interferon treatment compared to participants with no drug consumption (p<0.05). |
| Chang | 2022 | Taiwan | 2537 participants, mean age 51.9, SD 11.9 were recruited for a cross-sectional study in Taiwan from January 2015 to February 2018. | Participants who received interferon therapy did not have depression. |
| Huang | 2013 | Taiwan | 158 individuals treated with interferon therapy, of whom 73 had chronic hepatitis B, mean age 41, SD 9 years, were recruited for a prospective cohort study in Taiwan. | Participants with a treatment duration of more than 48 weeks, in comparison with less than 48 weeks, predicted more depression along treatment period (P=0.028).  Participants at 12 weeks of treatment predicted less depression (P=0.045). |
| Chong | 2018 | Taiwan | 48,215 participants, mean age 46, SD 15, were recruited for a retrospective population-based cohort study in Taiwan from 2000 to 2010. | There was no significant association between being on antiviral drugs for Hepatitis B infection and increased risk of subsequent bipolar disorder (HR 0.85, 95% CI 0.21–3.53). |

Abbreviations: SD, standard deviation; OR, odds ratio; CI, Confidence interval

†Outcomes of interest include logistic or linear regression analysis for any association between treatment factors and psychological outcomes

Supplementary Table 9: Evaluation of the mediating or confounding effect of income on psychological outcomes of Hepatitis B individuals

| **Author** | **Year** | **Country** | **Study population** | **Key findings†** |
| --- | --- | --- | --- | --- |
| Chan | 2012 | China | 149 individuals with a confirmed diagnosis of Hepatitis B, mean age 47.2, SD 11.9 years, were recruited for a cross-sectional study in China from 2008-2009. | Participants whose total family income was less than HKD$10,000 were more likely to have depressive disorders (p=0.003), but not anxiety disorders (p=0.07). |
| Daryani | 2008 | Iran | 100 Hepatitis B carriers, mean age 31.6, SD 11.6 years, were recruited for a cross-sectional study in Iran from 2004-2005. | Hepatitis B carriers with a monthly family income of 2.5 to 4 million rials (low income) were significantly more likely to have anxiety (p=<0.05) and abnormal psychiatric health (p=<0.01). |
| Liu | 2017 | China | 501,158 participants, of whom, 15558 had chronic hepatitis B were recruited for a cross-sectional study in China from 2004 to 2008. | Participants with hepatitis B with a lower income (10,000 to 35,000 yuan) were more strongly associated with depression (Odds ratio of 2.38 (95% CI: 1.34-4.25)). |
| Vu | 2019 | Vietnam | 298 participants, mean age 49.2, SD 16.0 were recruited for a cross-sectional study at the Chronic Hepatitis Clinic in the Viet-Tiep Hospital, Hai Phong, Vietnam. | Participants from the richest household income quintiles were associated with a lower likelihood of having depressive symptoms (OR = 0.06; 95%CI = 0.04;0.86). |
| Zhu | 2022 | USA | 313 Asian American participants, 177 not on antiviral medication, mean age 54, SD 13.42, and 136 on antiviral medication, mean age 53.08, SD 13.05, were recruited for a cross-sectional study in the United States between April 2019 and March 2020. | No significant relation was detected between income and risk for mild or severe depression, both among those not on medication (OR = 1.43, 95% CI 0.49-4.22) and those on medication (OR = 1.82, 95% CI 0.72-4.65). |

Abbreviations: SD, standard deviation; OR, Odds ratio; CI, Confidence interval

†Outcomes of interest include logistic or linear regression analysis for any association between income and psychological outcomes

Supplementary Table 10: Evaluation of the mediating or confounding effect of employment status on psychological outcomes of Hepatitis B individuals

| **Author** | **Year** | **Country** | **Study population** | **Key findings†** |
| --- | --- | --- | --- | --- |
| Zhu | 2022 | USA | 313 Asian American participants, 177 not on antiviral medication, mean age 54, SD 13.42, and 136 on antiviral medication, mean age 53.08, SD 13.05, were recruited for a cross-sectional study in the United States between April 2019 and March 2020. | Among participants on antiviral medication, being unemployed (OR = 4.53, p < 0.05) or not in the labor force (OR = 3.44, p < 0.05) were significant predictors of a higher risk for mild or severe depression. |
| Vu | 2019 | Vietnam | 298 participants, mean age 49.2, SD 16.0 were recruited for a cross-sectional study at the Chronic Hepatitis Clinic in the Viet-Tiep Hospital, Hai Phong, Vietnam. | Participants who were freelancers were associated with a lower likelihood of having depressive symptoms (OR = 0.39; 95%CI = 0.19;0.77). |
| Fotos | 2018 | Greece | 111 participants, mean age 44.85, SD 14.4, were recruited for a single center cross-sectional study in Greece from March to September of 2014. | Participants who were unemployed were significantly associated with higher levels of depression (p < 0.05). |

Abbreviations: SD, standard deviation; OR, Odds ratio; CI, Confidence interval

†Outcomes of interest include logistic or linear regression analysis for any association between employment status and psychological outcomes

Supplementary Table 11: Evaluation of the mediating or confounding effect of family history on psychological outcomes of Hepatitis B individuals

| **Author** | **Year** | **Country** | **Study population** | **Key findings†** |
| --- | --- | --- | --- | --- |
| Chan | 2012 | China | 149 individuals with a confirmed diagnosis of hepatitis B, mean age 47.2, SD 11.9, were recruited for a cross-sectional study in China from 2008-2009. | Individuals with current psychiatric (p<0.001), depressive (p<0.001), or anxiety disorders (p<0.001) were found to have a family history of mental illness. |
| Karlidag | 2019 | Turkey | 103 individuals with chronic hepatitis B treated with oral antivirals, mean age 39.0, SD 11.4 years, 94 untreated individuals with chronic hepatitis B, mean age 38.5, SD 12.1 years and 50 healthy individuals, mean age 36.6, SD 8.16, were recruited for a case-control study in Turkey in 2018. | For participants with a family history of chronic hepatitis B disease due to cirrhosis, anxiety (p=0.001) and depression (p=0.023) scores were determined to be significantly high. |
| Demir | 2013 | Turkey | 444 participants of which 249 HBsAg-positive inactive carriers (inactive carrier group) and 195 chronic hepatitis B individuals (chronic hepatitis group) that were undergoing follow-up were recruited for a cross-sectional study in Turkey. | There was no significant difference between anxiety levels and family history (P>0.05). |

Abbreviations: SD, standard deviation; OR, Odds ratio; CI, Confidence interval

†Outcomes of interest include logistic or linear regression analysis for any association between family history and psychological outcomes

Supplementary Table 12: Quality assessment of included cohort studies using the Joanna Brigg’s Institute Critical Appraisal tool

| **Study** | **1** | **2** | **3** | **4** | **5** | **6** | **7** | **8** | **9** | **10** | **11** |
| --- | --- | --- | --- | --- | --- | --- | --- | --- | --- | --- | --- |
| Chan | NA | NA | Y | Y | Y | NA | Y | NA | NA | NA | Y |
| Cortesi | Y | NA | Y | Y | Y | NA | Y | NA | NA | NA | Y |
| Daryani | NA | NA | Y | Y | Y | NA | Y | NA | NA | NA | Y |
| Karlidağ | Y | Y | Y | Y | Y | NA | Y | NA | NA | NA | Y |
| Gupta | NA | NA | Y | N | N | Y | Y | NA | NA | NA | Y |
| Huang 2019 | NA | NA | Y | Y | Y | Y | Y | NA | NA | NA | Y |
| Kiratli | NA | NA | Y | N | N | Y | Y | Y | Y | Y | Y |
| Y. Liu | NA | NA | Y | Y | Y | Y | Y | Y | Y | Y | Y |
| M. Liu | NA | NA | Y | Y | Y | Y | Y | NA | NA | NA | Y |
| Chong | Y | Y | Y | Y | Y | Y | Y | NA | NA | NA | Y |
| Marcellin | Y | Y | Y | N | N | Y | Y | Y | Y | Y | Y |
| Ngo | NA | NA | Y | Y | Y | NA | Y | NA | NA | NA | Y |
| Saffari | Y | Y | Y | Y | Y | NA | Y | NA | NA | NA | Y |
| Shaheen | NA | Y | Y | Y | Y | Y | Y | Y | Y | Y | Y |
| Vu | NA | NA | Y | Y | Y | NA | Y | NA | NA | NA | Y |
| Weinstein | NA | NA | Y | Y | Y | NA | Y | NA | NA | NA | Y |
| Yilmaz 2016 | Y | Y | Y | Y | Y | NA | Y | NA | NA | NA | Y |
| Yılmaz 2022 | NA | NA | Y | Y | Y | NA | Y | NA | NA | NA | Y |
| Zhu 2022 | NA | NA | Y | Y | Y | NA | Y | NA | NA | NA | Y |
| Ahmed | NA | NA | Y | Y | Y | Y | Y | NA | NA | NA | Y |
| Demir | Y | Y | Y | Y | Y | Y | Y | Y | Y | Y | Y |
| Alian | Y | Y | Y | Y | Y | Y | Y | NA | NA | NA | Y |
| Chang | NA | NA | Y | Y | Y | Y | Y | NA | NA | NA | Y |
| Cho | NA | NA | Y | Y | Y | Y | Y | Y | Y | Y | Y |
| Fotos | NA | NA | Y | Y | Y | Y | Y | NA | NA | NA | Y |
| Gale | NA | NA | Y | Y | Y | Y | Y | NA | NA | NA | Y |
| Huang 2013 | NA | NA | Y | Y | Y | Y | Y | NA | NA | NA | Y |
| Zhou | NA | NA | Y | Y | Y | Y | Y | NA | NA | NA | Y |
| Simonetti | Y | Y | Y | Y | Y | Y | Y | Y | Y | Y | Y |
| Kong | NA | NA | Y | Y | Y | Y | Y | NA | NA | NA | Y |
| Keskin | NA | NA | Y | Y | Y | Y | Y | NA | NA | NA | Y |

| Checklist |
| --- |
| 1. Were the two groups similar and recruited from the same population? |
| 2. Were the exposures measured similarly to assign people to both exposed and unexposed groups? |
| 3. Was the exposure measured in a valid and reliable way? |
| 4. Were confounding factors identified? |
| 5. Were strategies to deal with confounding factors stated? |
| 6. Were the groups/participants free of the outcome at the start of the study (or at the moment of exposure)? |
| 7. Were the outcomes measured in a valid and reliable way? |
| 8. Was the follow up time reported and sufficient to be long enough for outcomes to occur? |
| 9. Was follow up complete, and if not, were the reasons to loss to follow up described and explored? |
| 10. Were strategies to address incomplete follow up utilized? |
| 11. Was appropriate statistical analysis used? |

Legend:

Y – Yes

N – No

U – Unclear

NA – Not applicable

Supplementary Table 13: Comparison of anxiety and depressive symptoms among individuals of different chronic diseases

| **Author** | **Year** | **Country of study** | **Patient Characteristics** | **Prevalence of adverse mental health outcomes (%)** |
| --- | --- | --- | --- | --- |
| Maimaitituerxun^1^ | 2023 | China | Individuals with type 2 diabetes | 21.8% (Anxiety) |
| Mosleh^2^ | 2020 | Saudi Arabia | Chronic Kidney Disease individuals who are on hemodialysis | 19.7% anxiety  24.6% depression |
| Kačavenda-Babović^3^ | 2021 | Serbia | Individuals with chronic hepatitis C | 41% anxiety  24% depression |
| Younossi^4^ | 2015 | USA | Individuals with chronic hepatitis C | 24.5% depression |
| Tan^5^ | 2022 | Global | Individuals with hepatocellular carcinoma | 24.04% depression  22.20% anxiety |
| Camara^6^ | 2020 | Guinea | Individuals with HIV | 16.8% depression  13.8% anxiety |
| Duko^7^ | 2019 | South Ethiopia | Individuals with HIV | 32.0% depression  34.4% Anxiety |

*Prevalence

Supplementary Figure 1: Funnel plot for visual inspection of publication bias in studies assessing depressive symptoms prevalence in individuals with hepatitis B infection


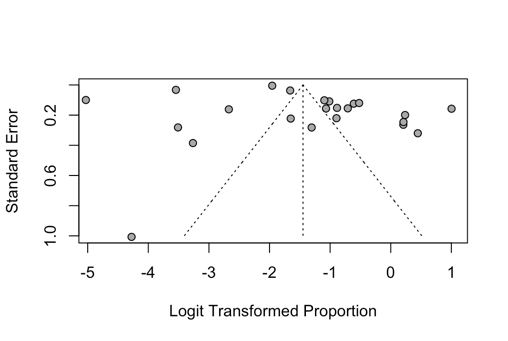


Supplementary Figure 2: Trim-and-fill analysis for publication bias in studies assessing depressive symptoms prevalence in individuals with hepatitis B infection


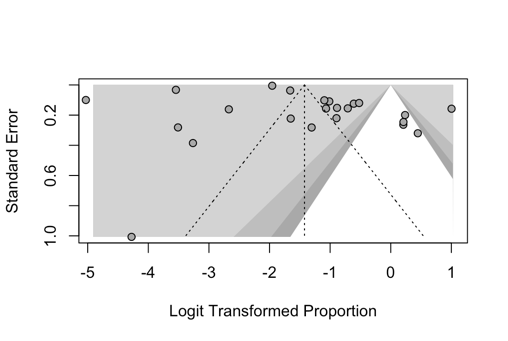


Supplementary Figure 3: Quantitative assessment publication bias in studies assessing depressive symptoms prevalence in individuals with hepatitis B infection


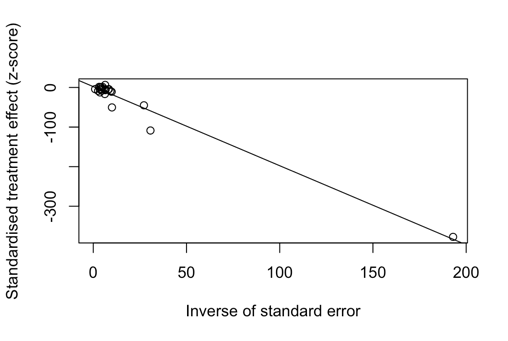


Supplementary Figure 4: Outlier assessment of studies assessing depressive symptoms prevalence in individuals with hepatitis B infection using the random effects model


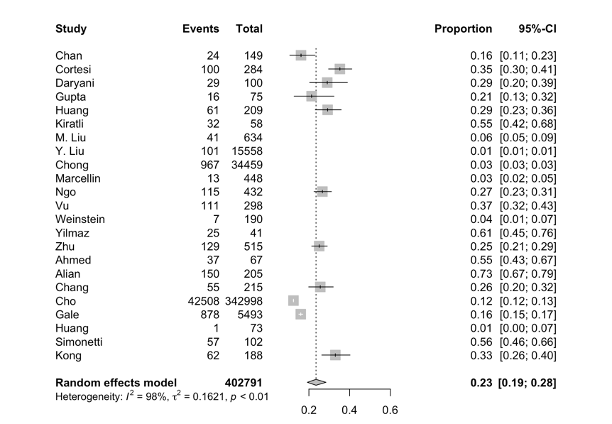


Supplementary Figure 5: Leave-one-out analysis of studies assessing depressive symptoms prevalence in individuals with hepatitis B infection using the random effects model


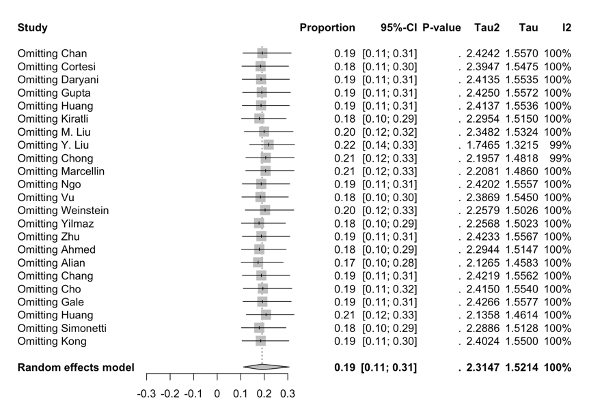


Supplementary Figure 6: Funnel plot for visual inspection of publication bias in studies assessing anxiety prevalence in individuals with hepatitis B infection


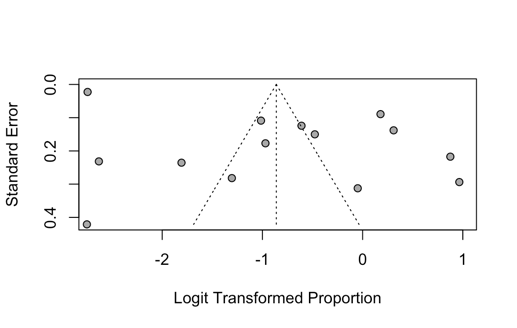


Supplementary Figure 7: Trim-and-fill analysis for publication bias in studies assessing anxiety prevalence in individuals with hepatitis B infection


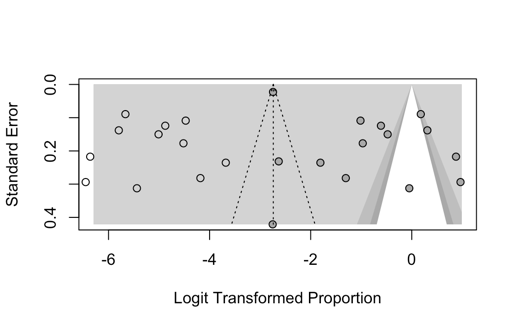


Supplementary Figure 8: Quantitative assessment publication bias in studies assessing anxiety prevalence in individuals with hepatitis B infection


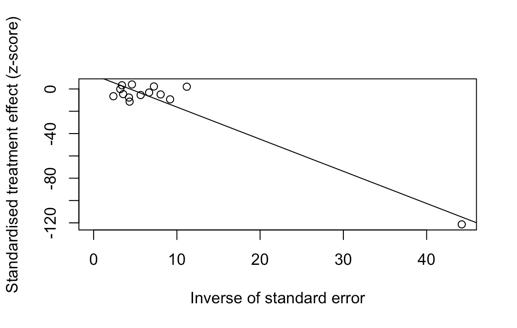


Supplementary Figure 9: Outlier assessment of studies assessing anxiety prevalence in individuals with hepatitis B infection using the random effects model


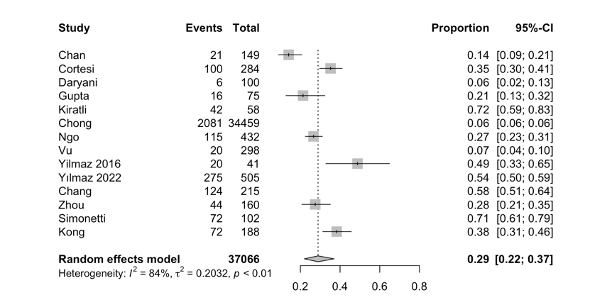


Supplementary Figure 10: Leave-one-out analysis of studies assessing anxiety prevalence in individuals with hepatitis B infection using the random effects model


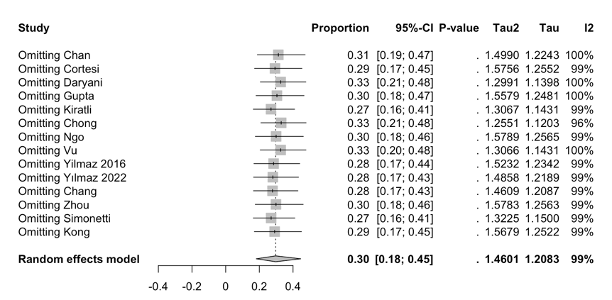


**References**

1. Maimaitituerxun R, Chen W, Xiang J, et al. Prevalence of Anxiety and Associated Factors Among Inindividuals with Type 2 Diabetes Mellitus in China: A Cross-Sectional Study. *Psychiatric Quarterly*. 2023/09/01 2023;94(3):371-383. doi:10.1007/s11126-023-10040-z

2. Mosleh H, Alenezi M, Al Johani S, Alsani A, Fairaq G, Bedaiwi R. Prevalence and Factors of Anxiety and Depression in Chronic Kidney Disease Individuals Undergoing Hemodialysis: A Cross-sectional Single-Center Study in Saudi Arabia. *Cureus*. Jan 15 2020;12(1):e6668. doi:10.7759/cureus.6668

3. Kačavenda-Babović D, Đurić P, Babović R, Fabri M, Ružić M, Bugarski Ignjatović V. DEPRESSION, ANXIETY AND QUALITY OF LIFE IN INDIVIDUALS WITH CHRONIC HEPATITIS C VIRUS INFECTION IN VOJVODINA. *Acta Clin Croat*. Dec 2021;60(4):579-589. doi:10.20471/acc.2021.60.04.03

4. Younossi ZM, Henry L, Park H, Adeyemi A, Stepanova M. Depression and Chronic Hepatitis C (CH-C): A Common and Costly Association: 2085. *Official journal of the American College of Gastroenterology | ACG*. 2015;110

5. Tan DJH, Quek SXZ, Yong JN, et al. Global prevalence of depression and anxiety in individuals with hepatocellular carcinoma: Systematic review and meta-analysis. *Clin Mol Hepatol*. Oct 2022;28(4):864-875. doi:10.3350/cmh.2022.0136

6. Camara A, Sow MS, Touré A, et al. Anxiety and depression among HIV individuals of the infectious disease department of Conakry University Hospital in 2018. *Epidemiol Infect*. Jan 14 2020;148:e8. doi:10.1017/s095026881900222x

7. Duko B, Toma A, Asnake S, Abraham Y. Depression, Anxiety and Their Correlates Among Individuals With HIV in South Ethiopia: An Institution-Based Cross-Sectional Study. Original Research. *Frontiers in Psychiatry*. 2019-May-07 2019;10doi:10.3389/fpsyt.2019.00290
